# Compliant sports floors and fall-related injuries: evidence from a residential care setting and updated meta-analysis for all patient care settings

**DOI:** 10.1136/ip-2022-044713

**Published:** 2022-12-23

**Authors:** Johanna Gustavsson, Finn Nilson, Carl Bonander

**Affiliations:** 1 Department of Political, Historical, Religious and Cultural Studies, Karlstad University, Karlstad, Sweden; 2 Centre for Public Safety, Karlstads Universitet, Karlstad, Sweden; 3 School of Public Health and Community Medicine, Institute of Medicine, Sahlgrenska Academy, University of Gothenburg, Gothenburg, Sweden

**Keywords:** hip facture, environmental modification, fall, non-randomized trial, outcome evaluation, older people

## Abstract

**Background:**

Compliant flooring may prevent fall injuries in residential care, but evidence is inconclusive. We investigate compliant sports floors and fall-related injuries in a residential care setting and update a meta-analysis from a recent systematic review on compliant flooring.

**Methods:**

A non-randomised study comparing outcomes in a residential care unit that installed sports flooring in bedrooms with four units with regular flooring in a Norwegian municipality (n=193). Data on falls were collected for a period of 46 months (323 falls on sports flooring; 414 on regular flooring). Outcomes were injurious falls per person bed-day, falls per person bed-day and injury risks per fall. Confounding was adjusted for using Andersen-Gill proportional hazards and log-binomial regression models. Random-effects inverse variance models were used to pool estimates.

**Results:**

Injurious fall rates were 13% lower in the unit with sports flooring (adjusted HR (aHR): 0.87 (95% CI: 0.55 to 1.37)). There was limited evidence of adverse effects on fall rates (aHR: 0.93 (95% CI: 0.63 to 1.38)) and the injury risk per fall was lower in fall events that occurred on sports floors (adjusted relative risk (RR): 0.75 (95% CI: 0.53 to 1.08)). Pooling these estimates with previous research added precision, but the overall pattern was the same (pooled RR for injurious falls: 0.66 (95% CI: 0.39 to 1.12); fall rates: 0.87 (95% CI: 0.68 to 1.12); injury risks per fall: 0.71 (95% CI: 0.52 to 0.97)).

**Conclusion:**

Sports floors may be an alternative to novel shock-absorbing floors in care settings; however, more research is needed to improve precision.

**WHAT IS ALREADY KNOWN ON THIS TOPIC**

- Preventing falls and fall-related injuries among older adults in residential care has proven difficult.
- Novel shock-absorbing flooring has been suggested as a passive fall injury-reducing measure.
- Compliant sports floor is a potential feasible option to novel shock-absorbing flooring, as it is widely available and comparable, although estimates are imprecise.

**WHAT THIS STUDY ADDS**

- The results from this study, combined with pooled data from a recent systematic review, suggest that sports flooring reduces the risk of fall injuries among older adults in residential care.
- Sports floors may have a similar injury risk-reducing potential as novel shock-absorbing floors.

**HOW THIS STUDY MIGHT AFFECT RESEARCH, PRACTICE OR POLICY**

- The current state of the evidence suggests that sports flooring can be considered for installation in residential care.
- Confirmatory randomised trials and updated cost-effectiveness analyses are warranted.

## Background

Older adults in residential care commonly have multiple fall risk factors.^1–3^ During a fall, the risk of injury depends on circumstance (eg, the type of fall), vulnerability (eg, bone strength), and—importantly—the energy-absorbing characteristics of the contact surface.^4–6^ Despite efforts to implement fall prevention programmes in residential care, their effectiveness remains uncertain.^7–9^ The lack of evidence-based approaches to preventing falls has led to the suggestion of installing compliant floors. By reducing energy transfer, compliant floors may help reduce the risk of injury during falls.

Laboratory studies have shown that compliant materials, including novel shock-absorbing floors, can reduce the amount of energy reaching human tissue,^10–13^ but clinical data are inconclusive. Several studies have shown a reduction in injury rates when novel shock-absorbing floors have been evaluated.^14–16^ However, a recent randomised controlled trial found no significant reductions in injury rates,^17^ raising doubts regarding the effectiveness of novel shock-absorbing floors and highlighting the need for studies of high methodological quality. Shock-absorbing floors may also have adverse effects on staff^18^ and are considerably more expensive than other types of flooring.

Sports floors with some compliant qualities (eg, 8.3 mm thick Tarkett Omnisports Excel) are cheaper alternatives^15^ that—according to a recent systematic review and meta-analysis of compliant flooring interventions^19^—may have comparable clinical effectiveness. Thus, they may provide similar injury-reducing effects at a lower cost, which has important implications for practice. However, the meta-analysis only contained two small-scale evaluations of sports floors,^19^ and the pooled estimates were imprecise. Therefore, additional research is needed to confirm if sports floors have similar effects on falls and injury rates as novel shock-absorbing floors.

The present paper presents new data on the effects of sports floors on falls and fall-related injuries in a Norwegian residential care facility and an updated meta-analysis comparing sports floors with novel shock-absorbing floors.

## Method

### Study design

In this non-randomised study, we aimed to evaluate the impact of installing compliant sports flooring (8.3 mm thick Tarkett Omnisports Excel) on the fall injury risk in a Norwegian residential care home setting. The intervention was initiated by the municipality and without randomisation.

### Setting and intervention

The study sites were all five residential care units in a Norwegian municipality, accommodating approximately 190 individuals. The units were high-level care homes, providing 24-hour access to help with activities of daily living and medical care. The evaluated flooring, an 8.3 mm vinyl floor over fibreglass mat with PVC foam backing, designed for sports venues (Tarkett Omnisports Excel (8.3 mm)), was installed in parts of one of the five units (forthwith called the intervention unit). The intervention unit had six wards and 72 single-room apartments. The sports flooring was installed in the bedrooms in the apartments. All other areas in the intervention unit—including bathrooms, corridors, etc—had standard flooring. The rest of the four care units included in the study had vinyl flooring with concrete underlay, creating a rigid flooring surface. These units were smaller with 16–36 apartments each, in total 109 apartments.

### Data collection

The data collection took place between May 2015 and March 2019. All residents living in the studied units were invited to participate, and written consent was collected. Participants were continuously recruited during the study period when moving in. The staff determined the resident’s capacity to consent based on their clinical expertise in evaluating cognitive ability. In cases where the resident could not give consent, the next of kin was asked to determine whether the resident was to participate. No participants moved out, but 57 participants passed away during data collection, and new residents who moved in were included in the study. Patient involvement was not applicable.

During data collection, all falls were registered following a previously existing on-site injury surveillance system. We defined a fall event as ‘an unexpected event in which the participants come to rest on the ground, floor or lower level’.^20^ For each fall event, information was recorded on time, date, location (bathroom, corridor, dining room/common area, bedroom/apartment), type of flooring (regular flooring or sports flooring), activity prior to fall (falls from sitting/lying height, falls when transferring between the bed and wheelchair, falls from standing height, falls when walking from and to bed, and unknown) and use of hip protector. Data on subsequent injuries included the injury outcome, diagnostic tools and treatment. The fall outcomes were categorised into ‘no injury’, ‘minor’, ‘moderate’ or ’severe’.^21^ When it was evident in the documentation of the event that the injury occurred in contact with something other than the flooring (eg, furniture, doorknob, etc), the event was excluded (n=5). In addition, we excluded 43 falls, of which 4 occurred outside, 10 occurred on other compliant surfaces (eg, mattresses or fall mats) and 29 were registered on a date outside the study period.

In conjunction with enrolment, the staff collected data on the participant’s age, sex, history of known falls during the last 12 months, and body height and weight from medical records. The following covariates were registered: medications (sedatives/tranquillisers/neuroleptics, antidepressants), sensory deficits (visual, hearing or motor impairment or no visual, hearing or motor impairment), cognitive impairment (cognitive impairment or no cognitive impairment) and walking ability (stable gait (with or without walking aid), unstable gait or unable to walk).

### Handling of missing data

Information on missing values can be found in table 1. For the statistical analyses, we imputed missing covariate data using multiple imputation chained equations with the *mice* package for R.^22^ We used predictive mean matching to impute continuous variables (n nearest neighbours=5), logistic regression for binary variables and polytomous regression for categorical variables with multiple categories (ie, the default settings in the *mice* package). We included all variables used in the analysis as predictors in the imputation model and generated 60 imputed datasets.^23^

**Table 1:** Descriptive statistics for all participants and fallers with at least one fall, intervention unit compared with control units

	All participants (n=193)			Fallers (n=136)		
	Unit with sports flooring	Unit with regular flooring	Missing (%)	Unit with sports flooring	Unit with regular flooring	Missing (%)
Participants	106	87		73	67	
Age at baseline (m (SD))	86.34 (7.30)	85.10 (9.06)	3.6	85.34 (7.68)	85.37 (8.34)	5.0
Women (n (%))	67 (63.8)	57 (66.3)	1.0	44 (61.1)	48 (71.6)	0.7
BMI (kg/m^2^) (m (SD))	24.40 (4.27)	23.49 (4.54)	29.5	25.16 (4.15)	23.73 (4.49)	30.0
Visual impairment (n (%))	54 (54.0)	46 (56.1)	5.7	37 (53.6)	32 (51.6)	6.4
Sedatives/tranquillisers/neuroleptics (n (%))	58 (56.9)	41 (50.0)	4.7	41 (58.6)	27 (43.5)	5.7
Antidepressants (n (%))	37 (36.3)	19 (23.2)	4.7	29 (41.4)	16 (25.8)	5.7
Cognitive impairment (n (%))	51 (51.0)	48 (64.0)	9.3	34 (50.0)	35 (63.6)	12.1
Walking ability (n (%))*			8.8			11.4
Safe walker	38 (37.6)	24 (32.0)		28 (40.6)	19 (34.5)	
Unsafe walker	30 (29.7)	40 (53.3)		26 (37.7)	32 (58.2)	
Non-walker	33 (32.7)	11 (14.7)		15 (21.7)	4 (7.3)	
History of falls (n (%))*	46 (48.9)	57 (72.2)	10.4	36 (55.4)	45 (76.3)	11.4
Fallen at least once during the study (n (%))	73 (68.9)	67 (77.0)	0	73 (100.0)	67 (100.0)	0
Number of falls (n, range, mean (SD))	323, 0–59, 3.05 (6.31)	414, 0–54, 4.76 (8.45)	0	323, 1–59, 4.42 (7.20)	414, 1–54, 6.18 (9.18)	0
Suffered a fall injury at least once during the study (n (%))	46 (43.4)	41 (47.1)	0	46 (63.0)	41 (61.2)	0
Number of fall injuries (n, range, mean (SD))	95, 0–16, 0.90 (1.89)	115, 0–17, 1.32 (2.37)	0	95, 0–16, 1.30 (2.16)	115, 0–17, 1.72 (2.58)	0
Bed-days (n, range, mean (SD))	90 735, 45–1460, 856 (520)	85 718, 62–1460, 985 (502)	0	65 837, 107–1460, 902 (517)	71 403, 161–1460, 1065 (435)	0

*Differs significantly (p<0.05) between intervention and control groups according to Χ^2^ tests.

BMI, body mass index.

### Outcomes

As recommended by Drahota *et al*,^19^ our primary outcome was injurious falls per person bed-day (any severity). Secondary outcomes included falls per person bed-day and injury risks per fall event (any severity).

### Statistical analysis

Descriptive statistics, in combination with Χ^2^ tests and t-tests, were used to assess the balance on baseline characteristics between intervention groups.

We used Andersen-Gill (AG) proportional hazards models^24^ to estimate unadjusted and adjusted HRs comparing injurious falls and falls per person bed-day between individuals living at the intervention site versus the four control sites. The AG method generalises traditional Cox regression to recurrent events, allowing us to include all events in the analysis. We assessed the proportional hazards assumption visually using unadjusted and adjusted Kaplan-Meier survival curves^25^ and tested for time-varying HRs using Schoenfeld residuals.^26 27^ In sensitivity analyses, we also fitted weighted Cox regressions that produce average HR in the presence of non-proportional hazards.^28^

The following covariates were included to adjust for potential confounding in the AG models: age, sex, body mass index (BMI), room type, time of day, activity type, cognitive ability, visual impairment, walking ability, history of falls, and the use of sedatives and antidepressants. This analysis aims to answer the question: ‘What is the impact of installing sports flooring in bedrooms on the overall fall and fall injury incidence in a residential care home setting?’. This question relates to the overall effectiveness of the intervention and is, for instance, most relevant for future economic analyses and decision-making. In a secondary analysis, we also split the AG analysis into events occurring in bedrooms (where sports flooring was installed) and those occurring elsewhere (where no sports flooring was installed).

We also used log-binomial regression analysis to estimate the association between sports flooring and injury risks per fall (n fallers=136, n falls=737). Log-binomial regression is an alternative to logistic regression that allows for direct estimation of the relative risk (RR).^29^ In this model, a fall event was considered treated if it occurred on sports flooring and untreated if it occurred on regular flooring. In addition to the individual-level covariates used in the AG models, this model also included the following event-specific variables: room type, time of day, activity type and hip protector use. This analysis aims to answer the question: ‘What is the injury-reducing effect of falling while walking on compliant flooring?’. This question relates to efficacy, that is, the effect of sports flooring in fall events that are actually exposed to the compliant surface.

All SEs account for individual clustering, and Rubin’s rules were applied to pool estimates and their variances from the imputed datasets.^30^ The statistical modelling was performed in R V.4.1.1.^31^

### Updated meta-analysis

To contextualise our results, we also updated a meta-analysis from a recent systematic review (figures 4, 6 and 9 in the SAFEST review^19^) with the primary estimates from the present study. Following Drahota *et al*,^19^ we used a random-effects inverse variance model^32^ with subgroups based on sports flooring (Tarkett Omnisports Excel in all included studies) and novel impact-absorbing flooring (Kradal or SmartCell in five included studies).

## Results

### Descriptive data on participants in the intervention unit versus control units

The study population consisted of 193 participants (124 women (64%)) (table 1), with a mean age of 85.3 (SD: 8) years. The period for data collection was 46 months, with 176 453 days of observed exposure time. Of the 193 participants, 73% (n=140) fell at least once. Participants’ baseline characteristics appear reasonably balanced between the intervention and control units (table 1), except for a significantly larger number of non-walkers and fewer residents with a history of falls in the intervention unit (all other comparisons are non-significant).

### Estimated effects of sports flooring on injurious fall events and falls

**Figure 1:**
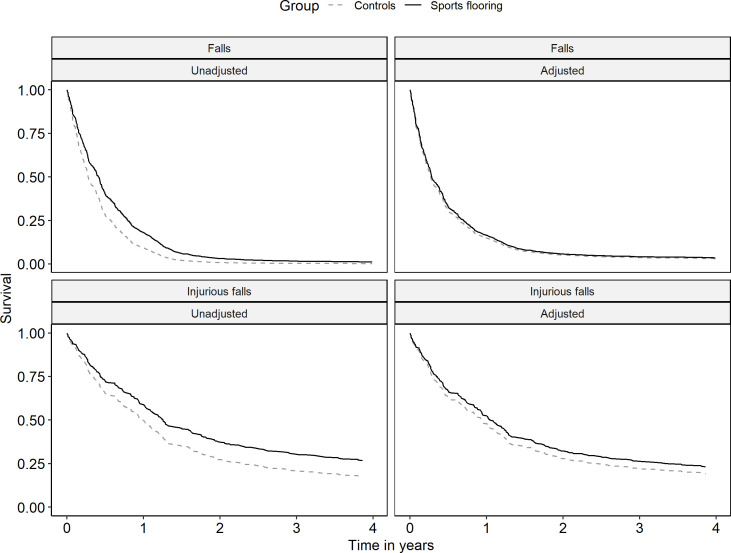
Unadjusted and adjusted Kaplan-Meier survival curves for falls and injurious falls in the intervention and control units. Adjusted curves include adjustment for age, sex, body mass index, room type, time of day, activity type, cognitive ability, visual impairment, walking ability, history of falls, the use of sedatives and antidepressants, room type, time of day, activity type and hip protector use.

Figure 1 shows unadjusted and adjusted survival curves for falls and injurious falls in the intervention unit and control sites.

The results from the AG model showed that the rate of injurious falls per person bed-day was 24% lower in the intervention unit (unadjusted HR 0.76, 95% CI: 0.44 to 1.30) compared with the control units, and 21% when adjusting for potential confounders (adjusted HR 0.87, 95% CI: 0.49 to 1.27) (table 2). There was no obvious visual indication of non-proportionality in the survival curves (figure 1), and the Schoenfeld residuals test was not significant in the adjusted model (p=0.27). Average HRs from the weighted models were almost identical (adjusted HR 0.88, 95% CI: 0.62 to 1.24). The difference in injurious fall rates was larger when restricting the analysis to events occurring in bedrooms (adjusted HR 0.80, 95% CI: 0.46 to 1.38) and closer to the null for events occurring elsewhere (adjusted HR 0.93, 95% CI: 0.56 to 1.54). The rate of falls per person bed-day was 28% lower in the intervention unit (unadjusted HR 0.72; 95% CI: 0.42 to 1.23), and 7% lower after confounding adjustment (adjusted HR 0.93; 95% CI: 0.63 to 1.38) (table 2). The survival curves for falls suggest that the differences in hazard between groups may be attenuated over time (figure 1). The residual test also indicates time-varying HRs for falls in the adjusted model (p=0.02). Consequently, applying a weighted model led to a quite different estimate of the average HR (adjusted HR 1.09, 95% CI: 0.78 to 1.51). The results for falls were similar for events occurring in bedrooms (adjusted HR 0.94, 95% CI: 0.62 to 1.44) and events occurring elsewhere (adjusted HR 0.91, 95% CI: 0.57 to 1.44).

**Table 2:** The risk of fall and injurious falls per person bed-day, intervention unit versus control units

	Intervention unit	Control units	HR, unadjusted	HR, adjusted
Fall events	323	414	0.72 (0.42 to 1.23)	0.93 (0.63 to 1.38)
Injurious events	95	115	0.76 (0.44 to 1.30)	0.87 (0.55 to 1.37)
Person bed-days	90 735	85 718		

HRs are based on Andersen-Gill proportional hazards models estimated using data from 193 individuals. Multivariable models are adjusted for age, sex, body mass index, room type, time of day, activity type, cognitive ability, visual impairment, walking ability, history of falls, and the use of sedatives and antidepressants. The 95% CIs that account for individual clustering and uncertainty from the imputation of missing data are shown in parentheses.

### Descriptive data on falls occurring on sports flooring versus regular flooring

We included 737 falls in our analysis; 164 occurred on sports flooring and 573 on regular flooring. Of these falls, 210 resulted in an injury (41 (25% of fall events) on sports flooring; 169 (29.5% of fall events) on regular flooring). Three out of four (74.7%) injuries with non-missing severity data (n missing=4) were classified as minor, 16.5% as moderate and 8% as severe. Only one injury resulted in death.

Mean age, BMI, visual impairment, use of sedatives/tranquillisers/neuroleptics and location were similar in falls that occurred on sports floors and regular floors. Still, the following characteristics differed significantly: sex, antidepressant users, individuals with cognitive impairment, walking ability, activity prior to fall, hip protector use, time of day and injury severity (table 3).

**Table 3:** Descriptive statistics for falls on sports flooring and regular flooring

	Sports flooring	Regular flooring	Missing (%)
n falls	164	573	
Age (m (SD))	87.28 (6.05)	86.49 (6.38)	2.8
Women (n (%))*	81 (49.7)	392 (68.4)	0.1
BMI (kg/m^2^) (m (SD))	24.42 (4.25)	24.60 (4.59)	25.1
Visual impairment (n (%))	68 (56.7)	309 (57.1)	10.3
Sedatives/tranquillisers/neuroleptics (n (%))	79 (50.3)	235 (42.1)	3.0
Antidepressants (n (%))*	84 (53.5)	176 (31.5)	3.0
Cognitive impairment (n (%))*	48 (41.7)	287 (65.8)	25.2
Walking ability (n (%))*			14.5
Safe walker	35 (29.4)	127 (24.9)	
Unsafe walker	61 (51.3)	338 (66.1)	
Non-walker	23 (19.3)	46 (9.0)	
Injured (n (%))	41 (25.0)	169 (29.5)	0.0
Injury severity (n (%))*			0.1
None	123 (75.0)	404 (70.6)	0.1
Minor	24 (14.6)	133 (23.3)	
Moderate	13 (7.9)	21 (3.7)	
Severe	4 (2.4)	13 (2.3)	
Death	0 (0.0)	1 (0.2)	
Location (n (%))			0.0
Bathroom	0 (0.0)	126 (22.0)	
Corridor	0 (0.0)	79 (13.8)	
Dining room/common area	0 (0.0)	147 (25.7)	
Bedroom/apartment	164 (100.0)	221 (38.6)	
Activity (n (%))*			0.0
Falls from sitting/lying height	11 (6.7)	35 (6.1)	
Falls when transferring between bed and wheelchair	28 (17.1)	92 (16.1)	
Falls from standing height	30 (18.3)	134 (23.4)	
Fall when walking from and to bed	39 (23.8)	86 (15.0)	
Unknown	56 (34.1)	226 (39.4)	
Hip protector (n (%))*	1 (4.2)	76 (31.3)	63.8
Time of day (n (%))*			
10:00–14:00	16 (9.8)	121 (21.1)	0.0
02:00–06:00	33 (20.1)	52 (9.1)	
14:00–18:00	25 (15.2)	139 (24.3)	
06:00–10:00	31 (18.9)	73 (12.7)	
18:00–22:00	21 (12.8)	115 (20.1)	
Unknown	38 (23.2)	73 (12.7)	

*Differs significantly (p<0.05) between falls on sports floors and falls on regular floors according to Χ^2^ tests.

BMI, body mass index.

### Estimated effects of falling on sports flooring versus regular flooring on injury risks

The unadjusted estimate from the log-binomial regression model showed an RR reduction of 15% per fall on sports flooring compared with regular flooring (RR 0.85, 95% CI: 0.63 to 1.14) and a 25% reduction for adjusted estimates (RR 0.75, 95% CI: 0.53 to 1.08). Expressed as a risk difference, these numbers correspond to −4.5 percentage points (unadjusted) and −7.5 percentage points (adjusted) (table 4).

**Table 4:** Log-binomial regression results for the effect of sports flooring on injury risk per fall

	Unadjusted	Adjusted
Relative risk	0.85 (0.63 to 1.14)	0.75 (0.53 to 1.08)
Risk per fall (sports flooring)	0.250	0.229
Risk per fall (regular flooring)	0.295	0.303
Risk difference	−0.045	−0.075

Results are based on log-binomial regression models estimated on 737 fall events among 136 individuals. Robust 95% CIs that account for individual clustering and uncertainty from the imputation of missing data are shown in parentheses for the relative risk estimates. The other estimates are based on predicted probabilities from the models. The multivariable model includes adjustment for age, sex, body mass index, room type, time of day, activity type, cognitive ability, visual impairment, walking ability, history of falls, the use of sedatives and antidepressants, room type, time of day, activity type and hip protector use.

### Updated meta-analysis

**Figure 2:**
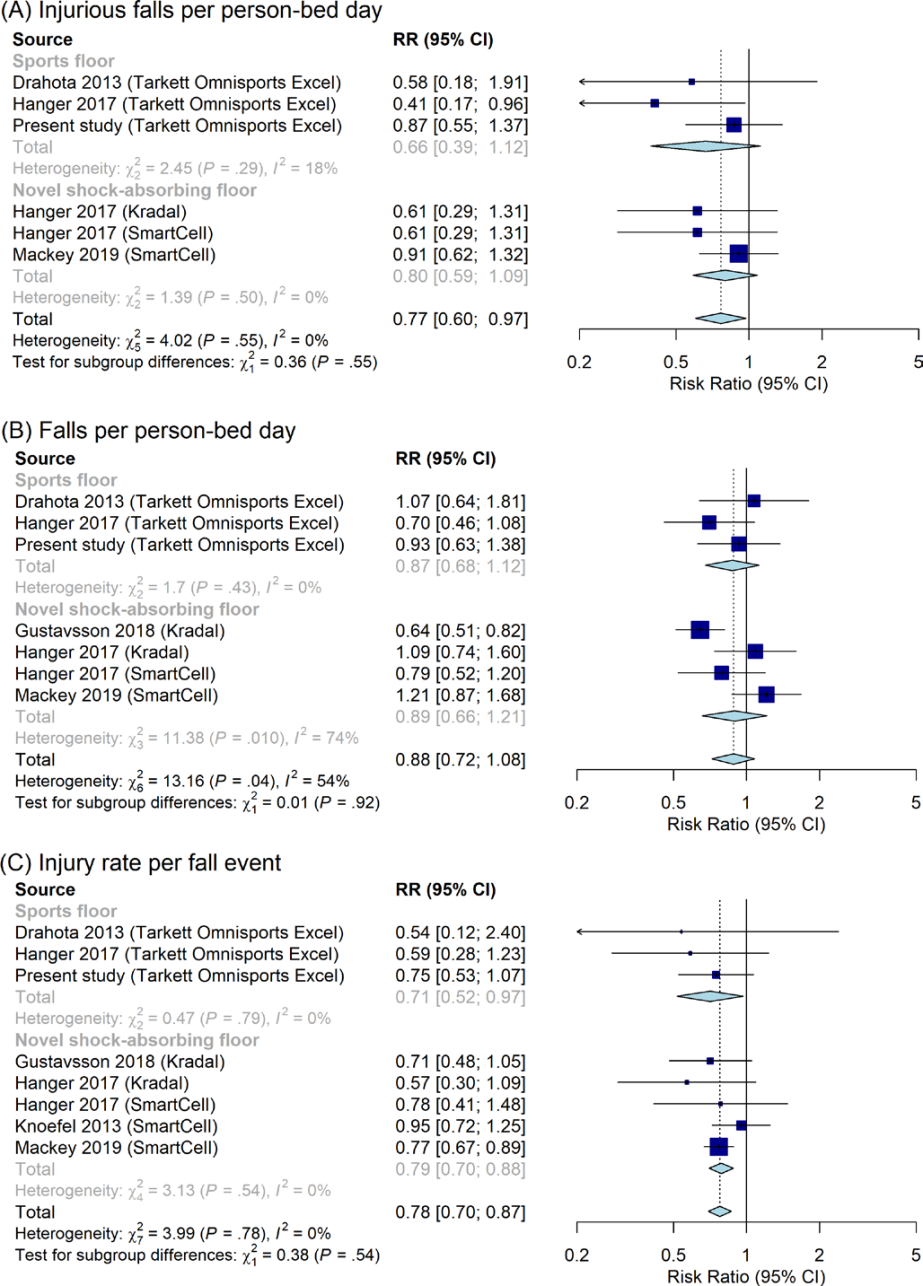
Updated random-effects inverse variance meta-analyses of studies evaluating the effect of compliant flooring versus regular flooring by subgroups defined by type of compliant flooring (sports floor or novel shock-absorbing floor). Outcomes: injurious falls per person bed-day (A), falls per person bed-day (B) and injury rate per fall event (C). All other estimates except those from the present study were extracted from figures 4, 6 and 9 of the SAFEST review.^19^ RR, relative risk.

Figure 2 shows the updated meta-analysis results including our primary HR estimates for sports flooring versus regular flooring on injurious falls per person bed-day (figure 2A), falls per person bed-day (figure 2B) and injury rates per fall event (figure 2C). Overall, the meta-analytical evidence suggests that the average RR for compliant flooring (of any type) is 0.77 (95% CI: 0.60 to 0.97) for injurious falls (figure 2A), 0.88 (95% CI: 0.72 to 1.08) for falls per person bed-day (figure 2B) and 0.78 (95% CI: 0.70 to 0.87) for injury rates per fall event (figure 2C). The subgroup-specific estimates for sports floors and shock-absorbing floors were only significant for the injury rates per fall outcome (figure 2A,B). Overall, the subgroup-specific estimates were similar in size except for injurious falls per person bed-day, which showed a larger point estimate for sports floors (RR=0.66) than shock-absorbing floors (RR=0.80). However, none of the subgroup differences were statistically significant.

## Discussion

Our results suggest that the sports flooring intervention may have an injury-reducing effect in a Norwegian residential care setting. However, our estimates are imprecise, and we cannot rule out that they are due to random chance. Indeed, the upper bounds of the 95% CIs are also consistent with a potential increase in risk. We therefore cannot make any conclusive statements about the effectiveness of sports floors in the studied setting. Some tendencies are, however, worth noting. First, our data showed a tendency towards lower risk of injurious falls in the intervention unit (−13%), with evidence of larger reductions for events occurring in areas where the sports floors were installed (ie, bedrooms: −20%). Second, the results for falls were inconsistent over time and were not sensitive to events occurring in bedrooms or elsewhere, suggesting that the differences we observed in fall rates may be explained by something other than the flooring intervention. Third, similar to Gustavsson *et al*,^14^ who investigated novel shock-absorbing floors in a Swedish nursing home, we find that falls occurring on sports floors were 25% less likely to lead to injuries than falls that occurred on other surfaces.

Adding our estimates to a recent meta-analysis that compared the effectiveness of sports floors with that of novel shock-absorbing floors,^19^ the combined state of the clinical evidence suggests that compliant floors can reduce the risk of injury. In terms of potential side-effects, our data and updated meta-analysis show no strong evidence that such flooring increases the risk of falling, which has historically been a concern.^33^ However, the possibility cannot be ruled out, as the upper bound CI of our meta-analysis implies that the evidence is consistent with both a decrease and an 8% increase (the upper bound potentially be used to inform worst-case scenarios in economic evaluations).

The theoretical expectation is that shock-absorbing floors—which are explicitly designed to reduce injury risks—would be more effective than sports floors. Such floors are specifically designed to have an injury-reducing function among older fallers rather than primarily being designed to be used in a sports setting. In terms of absorption, there are considerable differences between different floors^34^ and logically the higher the absorption capabilities, the better the fall injury-reducing effect. Nevertheless, the current clinical evidence does not support this theory and this study contributes to the idea that sports floors could be a reasonable alternative to the shock-absorbing floors that have been clinically evaluated so far. However, updated economic evaluations that compare sports floors with shock-absorbing floors are warranted.

### Strengths and limitations

Our study has several noteworthy limitations. First, treatment assignment was not under our control and not randomised. While we attempted to measure and adjust for suspected confounders, we cannot rule out the possibility that there are unobserved differences between the groups that influence the results. Second, treatment assignment was not blinded to the staff that recorded the fall and injury outcome data, so we cannot rule out potential bias from subjective reporting of injury outcomes. Third, it was not possible to measure time spent on each flooring type, and we could therefore not estimate incidence rates per time spent on sports or regular floors. Thus, our primary estimates reflect estimates of installing sports floors in bedrooms on overall fall and injury rates (ie, a reduced form estimate of the impact of sports floors). Fourth, the study is not powered to present results by injury severity. This is problematic as the most desired effect is a decrease in severe injuries. Fifth, our estimates are imprecise despite a lengthy data collection period. This problem is not unique to our study; it is also present in previous evaluations of sports floors.^16 35^ As recommended in a recent editorial in the American Statistician called ‘Moving to a World Beyond ‘p<0.05’’,^36^ we instead focused on ‘meta-analytical thinking’ when discussing the implications of our study. We hope others will consider doing the same in future studies to add more meta-analytical evidence on compliant floors since conducting sufficiently powered, large-scale studies on this topic is time-consuming and costly.

## Conclusions

Combined with previous research, our study suggests that sports floors may be a reasonable alternative to novel shock-absorbing floors as a fall injury prevention strategy in residential care homes. To our knowledge, there are also no reported adverse side-effects. While further research is needed to enhance precision in the comparison between flooring types, the data presented in this article can still be used as input for, for example, economic evaluations accounting for statistical uncertainty.

## Data Availability

Data are available upon reasonable request.
